# The Story of the Insane from Year to Year

**Published:** 1899-11-11

**Authors:** 


### THE STORY OF THE INSANE FROM YEAR TO YEAR.

(Eleventh Series.)

### THE LANCASHIRE ASYLUM AT RAINHILL.

Herein, on January 1st, 1897, were 1,838 patients, and by the end of the year this number had increased by 1 only. There were 270 first admissions, 30 readmissions, 101 recoveries, and 174 deaths. The recoveries stand at 33·66 per cent. of the admissions, and the deaths at 9·44 per cent. of the average number resident. Of the deaths during the year 57 were caused by cerebral and spinal diseases, 49 from consumption, 9 from pneumonia, 1 from enteritis, and 1 from typhoid fever. Of the year's admissions, 34 owed their insanity to various moral causes, 88 to intemperance in drink, and 46 to hereditary tendency. The asylum has been practically full during the year, and fresh cases could only be admitted as old cases were discharged or died. This is not a creditable state of things for any county to get into, more especially a rich and populous one like Lancashire; and as the annual increase in the number of the insane is a known one, there can be no difficulty in providing for that increase. Temporary buildings are being put up for 200 patients. Dr. Wiglesworth goes over the well-worn ground of the 4s. Government grant to the unions, and there cannot be the faintest doubt that this grant increased and increases the number of asylum patients. Nor is there any doubt that the poor old workhouse imbecile has a much better time in the asylum than he had in the workhouse. As to the alleged increase of insanity, Dr. Wiglesworth says, and we thoroughly agree with him, "that with the data at present at our disposal, the existence of an increasing tendency on the part of the population at large to mental breakdown can neither be definitely affirmed nor definitely denied. Were such a tendency proved to exist, however, it would create no astonishment, seeing how rampant in our midst are the causes operating in this direction." If an attendant, perhaps under provocation almost unbearable, strike a patient the assault soon assumes a legal aspect, and the newspapers invariably chronicle the "assault on a lunatic." When it is the lunatic who commits the assault we hear very little of the occurrence. In this report, however, Dr. Wiglesworth relates two serious assaults by patients on attendants. In one case the skull had to be trephined, and it was not considered that this attendant should remain at his work. As he had only been a short time in the asylum a gratuity, and not a pension, was given to him. The weekly charge to the unions was 8s. 2d. per head.

### THE LINCOLNSHIRE ASYLUM AT BRACEBRIDGE.

During the year 1897 the numbers fell from 757 to 710. There were 163 first admissions, 75 readmissions, 73 recoveries, and 90 deaths. The recoveries stand at the fair percentage of 38·2 on the admissions, and the deaths are high at 12·7 on the average number resident. Cerebral and spinal diseases caused death in 39 cases, consumption in 11, pneumonia in 2, and typhoid fever in 2. Of the year's admissions, the insanity was caused by various moral causes in 38 cases, by intemperance in drink in 26, and by hereditary tendency in 63. Among the year's admissions were 12 general paralytics, 18 idiots, 15 epileptics, and 31 were in a feeble and exhausted state from old age and disease—certainly not very promising material to deal with. In addition to these there were eight criminal patients. We have frequently remarked on the great harm done by these criminals in our county asylums, and we are pleased to quote Dr. Torney's views on the matter, which is really one requiring speedy attention at the hands of our Legislature. "These patients of the criminal class are," says Dr. Torney, "as a rule troublesome, and frequently are the cause of spreading disquietude and introducing vicious habits amongst the other patients. This class of patients should be placed under one control, and thereby many existing abuses would be done away with." There is no doubt that something should be done. Their number is a fairly constant one, and another criminal asylum should be built in some central position. There were six cases of typhoid fever, and of these two died, and there were also two deaths from enteritis. Dr. Dillon, the assistant medical officer, conducted classes for the nurses preparing for the St. John Ambulance examinations, and it was intended to continue the classes, and training the attendants and nurses for the Medico-Psychological examination. The staff may thus be trained step by step until they can obtain a three years' certificate from the Lincoln Asylum itself, and thus bring the nurses on a par with their hospital sisters. The weekly rate of maintenance is a trifle over 9s. a head.

### CHESHIRE COUNTY ASYLUM AT UPTON.

The number of patients in this asylum was 664 at the beginning of the year, and 734 at the end of it. There were 142 first admissions, 35 readmissions, 60 recoveries, and 86 deaths. Calculated on the admissions, the recoveries stand at 33·9 per cent., and the deaths, calculated on the average number resident, 12·3. The recoveries are below the county asylum average, and the deaths above it. Cerebral and spinal diseases are responsible for 38 of the deaths, consumption for 20, pneumonia for 4, and pleurisy for 2. In 22 of the year's admissions the insanity was caused by intemperance in drink, and in 36 by hereditary tendency. We have already remarked that the recovery rate was low and the death rate high, but all these statistics in asylums rise and fall with the nature of the year's admissions, and do not bear the significance that those ignorant of such institutions might think. When referring to the admissions, Dr. Lawrence points out that 11 were over seventy years of age, and that 17 did not live a month after admission. During the early months of 1897 the asylum was full, and hence the acute and, presumably, curable patients were not admitted in the usual numbers. Dr. Lawrence adds: "So long as there is accommodation and nursing facilities for such cases in the asylum, they are no doubt better looked after than they would be in most workhouses, but the fact of their being sent here exercises a considerable influence in producing what may be considered somewhat unfavourable statistics as regards the recovery and death rates." Post-mortem examinations were made in 66 of the 86 deaths, and Dr. Lawrence thinks this is a small proportion compared with some other asylums, while we are of opinion that it is unnecessarily large; but if Dr. Lawrence will tell us in his next report in how many cases he considered the post-mortem examination requisite to confirm his diagnosis, and in how many cases facts were brought to light the knowledge of which would improve the treatment of other cases, we should be in a better position to judge. Typhoid fever was imported during the year, but was happily limited to the first case, and with this exception there was no infectious disease. The annexe was opened during 1897, and the whole asylum was lighted by electricity. The assistant medical officers, Drs. Renton and Ambler, gave instructions to the attendants and nurses in first aid to the injured, and 43 passed the examination of the St. John Ambulance Association. This is an admirable beginning, and we should much like to hear that Dr. Lawrence has instituted a three years' certificate for his staff, and that the assistant medical officers have continued and extended their classes. The visitors say they have much satisfaction in again complimenting Dr. Lawrence and all the other officers on their efficient management of the institution. The charge to the Unions is 7s. per head per week.

